# ScaffComb: A Phenotype‐Based Framework for Drug Combination Virtual Screening in Large‐Scale Chemical Datasets

**DOI:** 10.1002/advs.202102092

**Published:** 2021-11-01

**Authors:** Zhaofeng Ye, Fengling Chen, Jiangyang Zeng, Juntao Gao, Michael Q. Zhang

**Affiliations:** ^1^ MOE Key Laboratory of Bioinformatics Bioinformatics Division Center for Synthetic and Systems Biology BNRist Department of Automation Tsinghua University Beijing 100084 China; ^2^ School of Medicine Tsinghua University Beijing 100084 China; ^3^ Center for Stem Cell Biology and Regenerative Medicine MOE Key Laboratory of Bioinformatics Tsinghua University Beijing 100084 China; ^4^ Tsinghua‐Peking Center for Life Sciences Beijing 100084 China; ^5^ Institute for Interdisciplinary Information Sciences Tsinghua University Beijing 100084 China; ^6^ Department of Biological Sciences Center for Systems Biology The University of Texas at Dallas Richardson TX 75080‐3021 USA

**Keywords:** deep learning, drug combination, scaffold, virtual screening

## Abstract

Combinational therapy is used for a long time in cancer treatment to overcome drug resistance related to monotherapy. Increased pharmacological data and the rapid development of deep learning methods have enabled the construction of models to predict and screen drug pairs. However, the size of drug libraries is restricted to hundreds to thousands of compounds. The ScaffComb framework, which aims to bridge the gaps in the virtual screening of drug combinations in large‐scale databases, is proposed here. Inspired by phenotype‐based drug design, ScaffComb integrates phenotypic information into molecular scaffolds, which can be used to screen the drug library and identify potent drug combinations. First, ScaffComb is validated using the US food and drug administration dataset and known drug combinations are successfully reidentified. Then, ScaffComb is applied to screen the ZINC and ChEMBL databases, which yield novel drug combinations and reveal an ability to discover new synergistic mechanisms. To our knowledge, ScaffComb is the first method to use phenotype‐based virtual screening of drug combinations in large‐scale chemical datasets.

## Graphical abstract

A phenotype‐based virtual screening pipeline of drug combinations in large‐scale chemical databases is proposed based on deep learning models. The phenotypic information is integrated into molecular scaffolds for drug screening. Screenings on the ZINC and ChEMBL databases demonstrate the potential of ScaffComb to identify potent drug combinations and novel synergistic mechanisms.

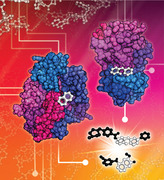

## 1 Introduction

Advances in our understanding of the molecular mechanisms of cancer biology have prompted the discovery of various drugs for cancer treatment. In recent decades, chemotherapy has become a practical option for modern cancer treatment.^[^ ^1^ ^]^ Despite the wide usage of anticancer drugs in clinical practice, monotherapeutic drug treatments face challenges such as drug resistance,^[^ ^2^, ^3^ ^]^ cancer relapse,^[^ ^4^ ^]^ low response rates,^[^ ^5^, ^6^ ^]^ and adverse side effects.^[^ ^7^, ^8^ ^]^ Therefore, combination therapy has been proposed as a promising solution.^[^ ^9^ ^]^ However, the efficient identification of potent drug combinations via computational prediction and experimental validation is still difficult, even with high‐throughput screening techniques.^[^ ^10^, ^11^ ^]^ Some of the difficulties lie in the exponential explosion of the large combinational number, the small number of effective drug combinations in all combinations^[^ ^12^ ^]^ and the complex synergistic mechanisms beneath the combinations.^[^ ^13^ ^]^ Fortunately, the increasing amount of pharmacological data has enabled the construction of large drug combination databases such as drug combination database (DCDB)^[^ ^14^ ^]^ and DrugComb,^[^ ^15^, ^16^ ^]^ which can discover new combinations and test new methodologies. Moreover, the emergence of powerful toolkits and algorithms in deep learning has led to several models with different neural network structures and input features. DeepSynergy^[^ ^17^ ^]^ was the first model to employ a fully connected network structure. Moreover, Xia et al.^[^ ^18^ ^]^ integrated multiomics data as input cell line features and combined different chemical descriptors as drug features. Furthermore, MatchMaker^[^ ^19^ ^]^ considers the order of drug pairs to design a branched network. However, all of these methods have been developed for relatively small chemical datasets of only several thousand compounds.

When applied to large‐scale databases such as ZINC and ChEMBL, drug combination screening can still be inefficient if we do not set restrictions when considering the underlying complex mechanisms. Conversely, phenotype‐based drug design^[^ ^20^ ^]^ uses phenotypic constraints for drug screening and is compatible with drug combination screening.^[^ ^20^, ^21^ ^]^ Moreover, phenotype‐based drug design does not depend on known protein targets but on effects such as cell viability and gene expression signatures. Nevertheless, methods enabling the use of phenotypes for virtual screening have yet to be explored. Méndez‐Lucio et al.^[^ ^22^ ^]^ introduced gene expression signatures as the condition in de novo drug design using generative adversarial networks (GAN), which incorporate phenotypic information into molecular structures. In addition, as scaffolds typically represent the core structures of compounds in analog series and chemical reactions, recently developed scaffold‐based methods^[^ ^23^ ^]^ allow us to screen drug libraries in terms of molecular scaffolds, which reduces the searching space.^[^ ^23^, ^24^ ^]^ Therefore, this study proposes a deep learning model that integrates phenotypic information into molecular scaffolds for drug screening.

In this work, we present ScaffComb, which is a phenotype‐based deep learning framework for the virtual screening of drug combinations in large‐scale chemical databases. We first describe the flowchart of ScaffComb and the details of two core modules in the framework: a generative module for integrating phenotypic information into drug screening (gene‐scaffold generator, GSG module) and a regression module for drug synergy prediction (simplified molecular‐input line‐entry system, SMILES‐based drug synergy predictor, SDSP module). We then validate ScaffComb by screening the US food and drug administration (FDA) dataset and reidentifying known drug combinations. In addition, we summarize drug synergy mechanisms with the screened FDA drug combinations, in which the combination of two molecularly targeted drugs is the most popular mechanism. Finally, we apply ScaffComb to large databases (ChEMBL and ZINC) to identify synergistic partners to known drugs or de novo drug combinations and synergistic mechanisms. Screening reveals correlations between phenotype specificity and the specificity of screened drug combinations. Furthermore, the results suggest that new synergistic mechanisms could be identified using general phenotypes. In summary, we demonstrate that ScaffComb is an effective tool for the virtual screening of drug combinations and inferring synergistic mechanisms.

## 2 Results

### 2.1 ScaffComb Deep Learning Framework for Drug Combination Prediction

**Figure 1:**
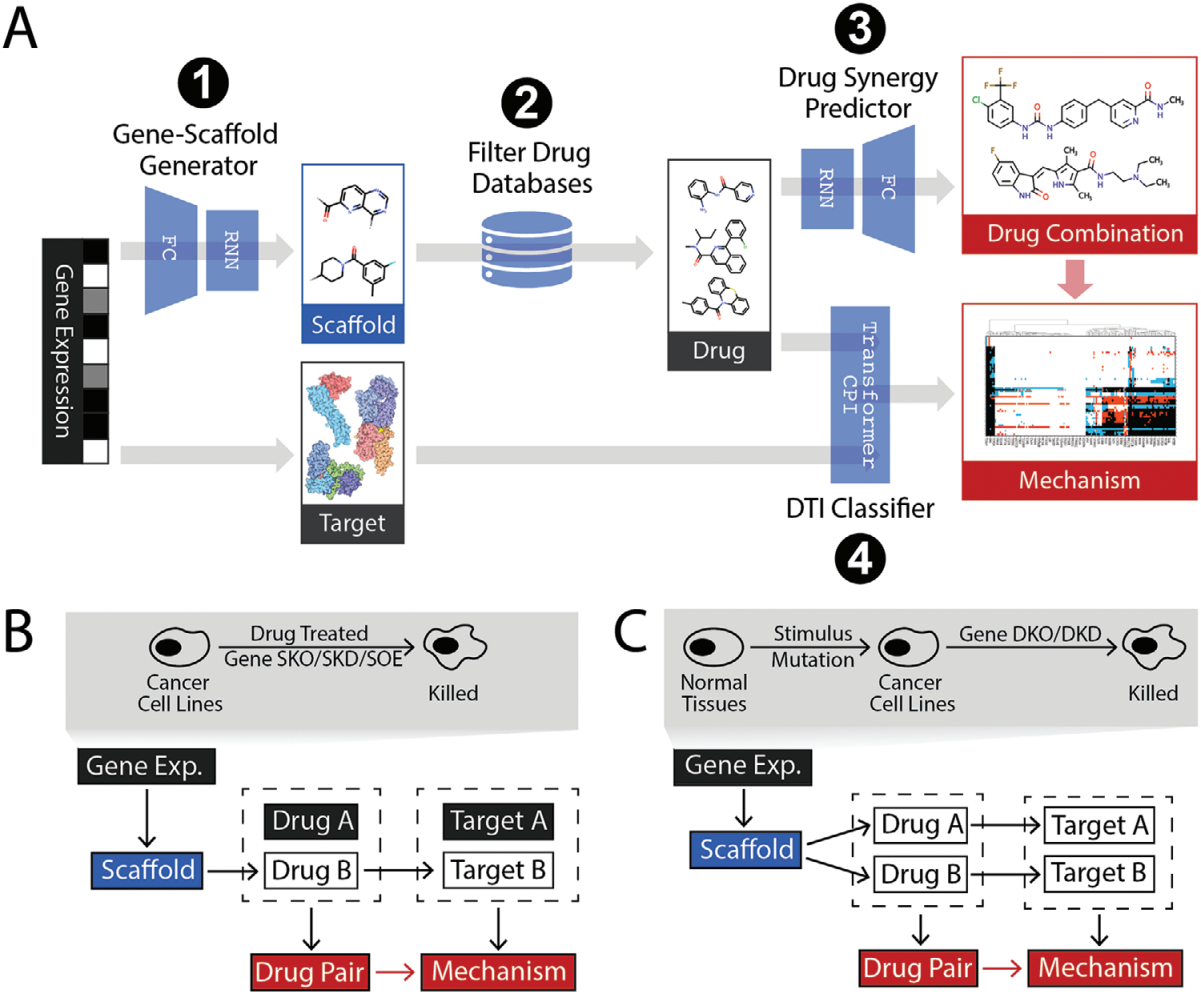
Overview of ScaffComb. A) Flowchart of ScaffComb. Four major steps are presented. Blue wedges indicate model blocks. Boxes indicate inputs (black), outputs (red), and intermediate items. FC: fully connected layers; RNN: recurrent neural network; DTI: drug‐target interaction. B,C) Two applications of ScaffComb. B) Identification of partner drugs to a known given phenotype. Drug B is screened and used to predict target B, combined with known targets of drug A for the combination mechanism. C) Screening of drug combinations from phenotypes, in which both drugs are screened and used to predict targets. Upper panels show typical input phenotypes for the applications. Lower panels show sketches of screening pipelines (black: inputs, red: outputs, white: intermediate items). SKO: single knockout; SKD: single knockdown; SOE: single overexpression; DKO: double knockout; DKD: double knockdown.

First, we provide a brief overview of the ScaffComb framework and its applications. ScaffComb includes three major deep learning models for screening drug combinations and inferring mechanisms (**Figure** **1**A and Figure S1, Supporting Information). 1) The GSG is a seq2seq model with an attention mechanism.^[^ ^25^ ^]^ 2) The drug synergy predictor (DSP), a regression model, processes the drug and cell line features for drug combination synergy score prediction. 3) The drug‐target interaction (DTI) predictor (TransformerCPI^[^ ^26^ ^]^) takes a transformer framework and uses the drug SMILES and protein amino acid sequences to predict DTIs.

The general flowchart of ScaffComb is as follows. 1) The GSG uses phenotypes as inputs to generate scaffolds. The phenotypes employed in this work are differentially expressed genes (DEGs) between two cell states (e.g., drug‐treated vs untreated), which are encoded as a vector composed of values in {−1,0,1} space representing downregulated DEGs, non‐DEGs, and upregulated DEGs, respectively (details are provided in the Experimental Section). 2) Scaffolds are filtered and used to screen a given chemical database, such as ZINC and ChEMBL. 3) The filtered compounds in (2) are either combined with a given drug (Figure 1B) or compounds with other scaffolds (Figure 1C) to obtain drug pairs. Drug pairs are then submitted to the SDSP to identify the most synergistic drug combinations. 4) A list of DEG products (proteins) in (1) is considered a reference target for inferring synergistic mechanisms. Specifically, the downregulated DEGs in perturbed cancer cell lines (Figure 1B upper panel) and upregulated DEGs in cancer cells to normal cells (Figure 1C upper panel) were considered in this study. Drugs in candidate drug combinations and reference targets were submitted to the DTI classifier to identify target proteins. In this study, we used TransformerCPI^[^ ^26^ ^]^ to predict the DTIs. The BindingDB database was preprocessed and used to train the TransformerCPI. Finally, the targets of the two drugs in combination were compared and annotated to infer the potential drug synergy mechanisms.

Two general applications of ScaffComb are demonstrated in this study. The first involves predicting the synergistic partner of a known drug with a given phenotype (Figure 1B). The commonly used phenotypes can be DEGs between basal and single‐gene perturbed (knockdown, knockout, and overexpression) or drug‐treated cell lines. Second, de novo drug combinations based on the phenotype input can be carried out (Figure 1C). We used two types of phenotypes as examples of this application. DEGs in cancer cell lines to normal cells represent general phenotypes, which are hypothesized to have broad synergy strategies. In contrast, DEGs of gene double knockouts (DKOs) in cancer cell lines represent target‐specific phenotypes, which are restricted to a narrower range of synergistic mechanisms. These phenotypes provide biological insights that drive the screening process. Before describing the specific applications and cases, it was necessary to better understand the operation and performance of the core modules, that is, GSG and SDSP.

### 2.2 GSG Module for Embedding Phenotypic Information into Chemical Structures for Drug Screening

The first step in successful drug screening is to set reasonable constraints, of which phenotypes are one of the most commonly used. Researchers have been interested in bridging the gap between phenotypes and chemistry, with several studies integrating phenotypic (genomic) and chemical spaces in DTI predictions.^[^ ^27^, ^28^, ^29^ ^]^ For example, by mapping both spaces to the pharmacological space, distances can be directly measured to infer DTIs.^[^ ^23^ ^]^ In a recent study by Méndez‐Lucio et al.,^[^ ^22^ ^]^ drug generation processes were coupled with phenotypes as the conditions in a conditional GAN ^[^ ^30^ ^]^ model using L1000^[^ ^31^ ^]^ drug perturbation data. However, this model suffers from a low generative validity ratio of molecules^[^ ^22^, ^32^ ^]^ and cannot be applied directly to drug screening. Therefore, by introducing molecular scaffolds and SMILES^[^ ^33^ ^]^‐based recurrent neural networks (RNNs),^[^ ^34^ ^]^ a GSG model was proposed to embed phenotypic information into scaffold structures for drug screening.

The GSG model is composed of an encoder and an attention decoder. The encoder processes DEG vectors to the context vectors, and the decoder generates the SMILES of the scaffolds (Figure S1A, Supporting Information). The stack‐augmented long short‐term memory (SA‐LSTM)^[^ ^35^ ^]^ neural network is used in the decoder to better learn the SMILES rules and enhance the validity ratio of the structure generation.^[^ ^36^ ^]^ In this study, GSG used discrete DEG sequences of L1000 landmark genes as the inputs, which represent a vector of size 978 (see the Experimental Section for more details). The gene vectors were composed of values in {−1,0,1} space, in which −1, 0, and 1 represent downregulated DEGs, non‐DEGs, or upregulated DEGs in L1000 landmark gene sets, respectively. Furthermore, the processing of drugs to scaffolds followed retrosynthetic combinatorial analysis procedure (RECAP) rules,^[^ ^37^ ^]^ which are commonly used in small molecule fragmentation in terms of the 11 types of chemical bond breakage (see the Experimental Section). A dataset of gene vector‐scaffold pairs was constructed from L1000 drug perturbation data as the training and validation sets (see the Experimental Section). We used a two‐step training schedule for scaffold generation. The stack‐augmented LSTM of the decoder was first trained with ChEMBL scaffolds to learn the basic rules for generating valid scaffolds. Then, the whole model was trained with L1000 samples that contained the scaffolds and gene vectors. The teacher‐forcing method^[^ ^38^ ^]^ was used during training. We then validated the performance of the model using the test set and the L1000 gene knockdown data.

**Figure 2:**
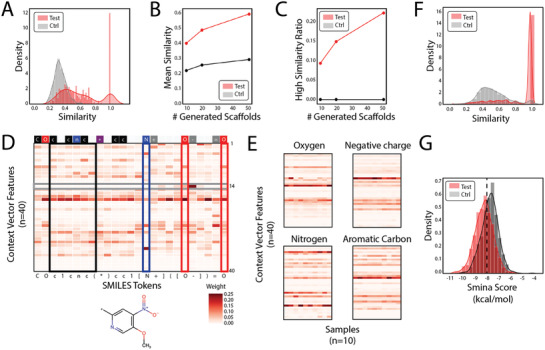
Performance validation of the gene‐scaffold generator. A) Tanimoto similarity between label scaffolds and generated scaffolds (red) or randomly sampled scaffolds (gray) in the test set. B) Mean similarity and C) ratio of scaffolds with a high similarity change with the number of scaffolds generated. D) An example attention weight heatmap of scaffold SMILES tokens and context vector features. Boxes indicate several patterns. Black: aromatic atoms; blue: nitrogen atoms; red: oxygen atoms; gray: feature 14. Upper bar shows types of tokens. Red: oxygen atoms; blue: nitrogen atoms; black: carbon atoms; gray: charge or bond tokens; purple: breakpoint token; white: other structural tokens. E) Feature patterns of oxygen atoms, negative charges, nitrogen atoms, and aromatic carbon atoms. Context vector features of these tokens from ten samples were displayed. F) Overall similarity between inhibitor scaffolds and generated scaffolds (red) or randomly sampled scaffolds (gray). G) Distribution of binding affinities (smina score) of screened drugs (red) and randomly chosen drugs (gray) to the JAK3 protein kinase domain.

First, we sampled 2000 DEG vectors in the test set and generated 10, 20, or 50 scaffolds for each sample. We randomly selected the same number of ChEMBL scaffolds as the control. The distribution of Tanimoto similarity^[^ ^39^ ^]^ between the label scaffolds and the generated scaffolds in contrast to the control scaffolds when 50 scaffolds were used for comparison is presented in **Figure** **2**A. The mean similarity (Figure 2B) and high similarity scaffold ratio (Figure 2C, similarity ≥0.8) increased much faster than the control with an increasing number of generated scaffolds in the test set. In addition, we checked the attention weights, which showed the importance of features in context vectors to the SMILES tokens in the scaffolds (see Figure 2D for an example). The aromatic atoms exhibited similar patterns, which were quite different from patterns of oxygen and nitrogen atoms (Figure 2E). Feature 14 was activated explicitly in negative charges (Figure 2D,E). Furthermore, we used the integrated gradient^[^ ^40^ ^]^ to check the input contributions to scaffold generation (Figure S2A, Supporting Information). As expected, the upregulated and downregulated genes contributed to scaffold generation. These results show that GSG can learn to map DEG information into context vectors, which is responsible for the generation of distinct scaffold structures.

We further validated the performance of GSG on gene knockdown samples from the L1000 database. The primary assumption is that, if GSG can incorporate gene knockdown information, the generated scaffolds should be similar to the known inhibitors of these genes. Therefore, we used 4287 gene knockdown samples from L1000, found the corresponding inhibitors (IC_50_ ≤ 1 × 10^−6^ m) in ChEMBL databases, and generated 20 scaffolds for each inhibitor. The primary relationship between the intratarget inhibitor similarity and the number of inhibitors is shown in Figure S2B in the Supporting Information. For most genes, the intratarget similarity was relatively low, reflecting some diversity in the inhibitor scaffolds. Overall, the generated scaffolds showed significantly higher similarity to the inhibitor scaffolds than the randomly sampled scaffolds (Figure 2F). The same results were true when viewing from the cell line perspective (Figure S2C, Supporting Information).

Moreover, we used the generated scaffolds from the JAK3‐KO gene signature to screen the ChEMBL database. The general screening flowchart is shown in Figure S3A in the Supporting Information. In the screening, we successfully identified several known inhibitors (Figure S3B, Supporting Information) and similar compounds (Figure S3C, Supporting Information) to known JAK3 inhibitors. In general, the screened compounds were much more similar to the known inhibitors than the randomly chosen ChEMBL compounds (Figure S3D, Supporting Information). We further used smina^[^ ^41^ ^]^ to dock the screened compounds to the JAK3 protein kinase domain (Figure S3E, Supporting Information, protein data bank (PDB) ID: 5TOZ). The distribution of docking scores showed stronger binding affinities of the screened drugs to JAK3 than randomly chosen control drugs (Figure 2G). This result provides a good demonstration of phenotype‐based virtual screening using GSG and indicates that GSG can effectively integrate gene expression signatures into scaffold structures and facilitate downstream drug screening.

### 2.3 SDSP Module with SMILES‐Based Features to Improve Drug Synergy Predictions

**Figure 3:**
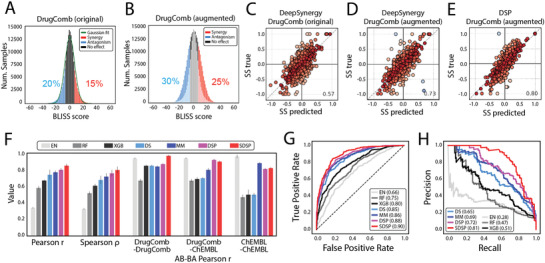
Data augmentation and performance estimation for SMILES‐based drug synergy predictor. A) Data distribution of Bliss synergy scores in original DrugComb samples and B) augmented samples. C–E) Effects of data augmentation and structure modification on the synergy score prediction. Pearson correlation coefficient between true values and predictions. Colors show the difference. Warmer colors indicate minor differences, whereas colder colors indicate significant differences. C) Original DrugComb samples with DeepSynergy (fully connected network). D) Augmented DrugComb samples with DeepSynergy. E) Augmented DrugComb samples with DSP. F) Synergy score predictor performance in the test set. Pearson correlation, Spearman correlation, and AB‐BA Pearson correlation between predictions and labels were compared across different methods. DrugComb‐DrugComb: both drugs were sampled from the DrugComb dataset. DrugComb‐ChEMBL: one drug was sampled from the DrugComb dataset and the other was sampled from the ChEMBL database. ChEMBL‐ChEMBL: both drugs were sampled from the ChEMBL database. G,H) receiver operating characteristic (ROC) and precision‐recall curve (PRC) curves for synergy classification across different methods in the test set. The area under the receiver operating characteristic (AUROC) and the area under the precision‐recall curve (AUPRC) are listed in the boxes. EN: elastic net; RF: random forest; XGB: XGBoost; DS: DeepSynergy; MM: MatchMaker; DSP: drug synergy predictor; SDSP: SMILES‐based drug synergy predictor.

Regarding the screening modules, the next thing to consider is how to score and identify potent drug combinations. Therefore, synergy score prediction is also vital for identifying synergistic drug combinations in cell lines. In ScaffComb, we used SMILES‐based drug feature extraction along with data augmentation to build and train the SDSP. The inputs of SDSP consist of a basal expression signature of L1000 landmark genes as cell line features and randomized SMILES strings as drug features. Randomized SMILES strings represent a drug by randomly selecting SMILES strings from all viable SMILES strings representing this chemical structure. Previous research has shown that randomized SMILES strings can improve feature extraction and the generalizability of RNNs compared to canonical SMILES strings.^[^ ^42^, ^43^ ^]^ We used the DrugComb dataset^[^ ^15^ ^]^ to train and test SDSP. As shown in **Figure** **3**A, the distribution of synergy scores in the dataset was close to a normal distribution. However, there were fewer samples with relatively high synergistic effects and with antagonistic effects than ones without apparent effects (Figure 3A). The former two types of sample were of interest in this study. Therefore, we used a binwise oversampling of samples in the original histogram (Figure 3B, see the Experimental Section) to balance the three types of samples in the training set. Using the augmented dataset (Figure 3C,D), a better prediction performance was achieved in the test set than in the original dataset (Pearson's *r*: 0.73 vs 0.57). The structure of SDSP is shown in Figure S1B in the Supporting Information, which contains individual encoders for drug and cell line feature processing and two fully connected networks for interaction predictions, considering that the order of the drugs in a sample should not affect predictions. In addition, a loss that measured the differences between the two predictions was added to the total loss as regularization and the mean of the two synergy scores was used as the final prediction (see the Experimental Section). This structure further improved the prediction performance (Figure 3D,E, Pearson's *r*: 0.80).

We compared SDSP with three baseline methods and two deep learning models (DeepSynergy^[^ ^17^ ^]^ and MatchMaker^[^ ^19^ ^]^) that use chemical fingerprints as the drug features. We also built a DSP for better comparison, which shared a similar structure with SDSP but used chemical fingerprints as drug features instead of SMILES. The results showed that SDSP generally outperformed other methods and achieved state‐of‐the‐art performance in both regression and classification situations (Table S1, Supporting Information, and Figure 3F–H).

In addition, we checked the Pearson *r* between the predicted synergy scores in a sample (drug A‐drug B‐cell line features) and its corresponding drug‐order‐reversed sample (drug B‐drug A‐cell line features), which we call the AB‐BA correlation in this study. The AB‐BA correlation is used to illustrate the biases of how models process drug features. For comparison, 1000 AB‐BA correlations of drug combinations constructed from three different sources were calculated using diverse methods: (1) combinations of two DrugComb drugs sampled from the test set (DrugComb‐DrugComb) represented samples that were closest to the training samples; (2) combinations of a DrugComb drug and a ChEMBL drug represented mixed samples with partially unseen chemical structures; and (3) combinations of two ChEMBL drugs represented unseen samples (Figure 3F and Table S1 and Figure S4, Supporting Information). Clearly, SDSP, DSP, and MatchMaker were more stable when dealing with unseen compounds. However, although the elastic net was not influenced by the drug feature order, it predicted small values for all samples (Figure S4, Supporting Information).

Finally, we validated the influence of randomized SMILES strings. We first trained another SDSP model using the DrugComb dataset with canonical SMILES strings (SDSP‐can). We then sampled 1000 drug combinations. For drugs in each combination, ten randomized SMILES strings were generated using RDKit,^[^ ^44^ ^]^ which yielded 100 samples. Synergy scores were calculated across samples, and the full width at half maximum (FWHM) of the synergy score distribution was measured. Consequently, SDSP had a narrower FWHM distribution than SDSP‐can and randomly chosen controls for drug combinations sampled either from DrugComb (Figure S5A, Supporting Information) or ChEMBL (Figure S5B, Supporting Information). This suggested that SDSP had learned to recognize different SMILES forms of the same drug. Figure S5C in the Supporting Information shows an example of the randomized SMILES synergy score distribution across cell lines. These results indicate that SDSP can better process chemical features and make reasonable predictions of drug combination synergy scores.

### 2.4 Reidentifying Known FDA Drug Combinations with ScaffComb during Validation Screening

**Figure 4:**
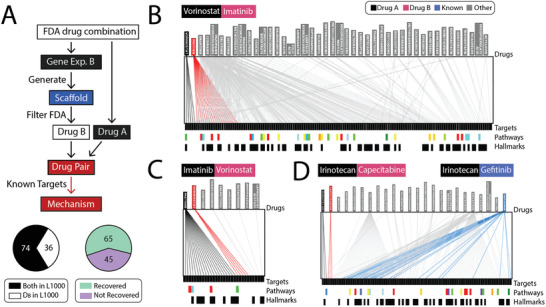
Combined drug screening in the FDA‐approved drug dataset. A) Flowchart of ScaffComb screening in the FDA dataset. Lower left panel shows sample types and lower right panel shows the ratio of reidentified drug combinations using ScaffComb. D_B_: perturbed drugs whose DEG vectors were used as phenotypes in screening. B,C) Target overlapping of vorinostat, imatinib, and screened drugs with: B) the imatinib perturbed DEG vector used as the phenotype and C) the vorinostat perturbed DEG vector used as the phenotype. D) Another known combined drug of irinotecan (black), gefitinib (blue), was screened using capecitabine DEG vector as the phenotype. Upper boxes show drug combination components. Lower color bars show different enriched pathways. Lower black bars represent hallmark genes in cancer.

To illustrate the practical applications of ScaffComb, we validated ScaffComb using the FDA‐approved drug dataset, which is the gold standard dataset. Our goal was to determine whether ScaffComb can reidentify known drug combinations in the FDA dataset. The screening flowchart is shown in **Figure** **4**A. We retained FDA drug combinations in which at least one of the drugs had perturbed gene expression signatures in the L1000 dataset. A total of 110 FDA drug combinations were filtered to validate ScaffComb (see the Supporting Information for more information). For 74 of the drug combinations, the perturbed gene expression signatures were available for both drugs, whereas the other 36 combinations had gene expression signatures for one of the drugs (Figure 4A). The general procedure is summarized in Figure 4A. First, DEG vectors for drug B were used to generate 100 scaffolds. Next, these scaffolds were filtered and used to screen FDA datasets. Then, the filtered drugs were combined with drug A to calculate the synergy scores using SDSP. Finally, the targets of drugs in potent combinations (synergy score ≥5 or synergy score ≤ −5) were obtained from ChEMBL, which were filtered with L1000 best‐inferred targets (see the Experimental Section for more information). As shown in Figure 4A, screening successfully identified 65 out of 110 known combinations. In the combination of vorinostat and imatinib, the two drugs targeted different proteins and pathways, whereas imatinib had broader targets across kinases. By employing the DEG of imatinib for screening, we consistently identified drugs with broader mechanisms (Figure 4B) and vice versa (Figure 4C). The mechanism selection reflected phenotype constraints during screening. Furthermore, an alternative mechanism was identified in the irinotecan capecitabine combination screening, which corresponds to another known combinable drug, gefitinib (Figure 4D). This implied that the same phenotype could result from different mechanisms, suggesting that ScaffComb could identify potential drug combinations in diverse mechanisms. More cases can be found in Figure S6 in the Supporting Information, and more detailed information on these figures can be found in the Supporting Information.

**Figure 5:**
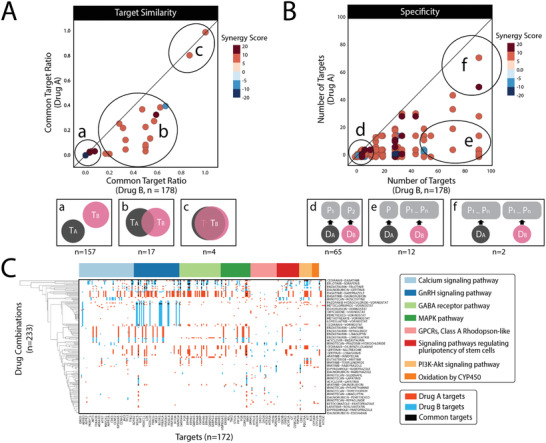
Combination mechanism of FDA drugs. A–C) Drug combination mechanism in terms of A) target similarity, B) drug specificity, and C) target pathways. A,B) Lower panels (a–g) show inferred illustrations of the mechanisms corresponding to the upper panels. The number of samples in each type is shown below. T represents the target, D represents the drug, and P represents the protein. Color bar shows synergy scores in the scatterplots. C) Drug combination pathway heatmap. Columns represent drug targets. Rows represent FDA drug combinations. Red indicates the targets of drug A, blue indicates the targets of drug B, and black indicates common targets. Top color bar presents enriched gene ontology annotations.

Furthermore, most FDA‐approved drugs have known targets, which enable us to systematically analyze combination mechanisms. Therefore, we checked the mechanisms of all screened FDA drug combinations from the perspective of target similarity (**Figure** **5**A), drug specificity (Figure 5B), and target pathways (Figure 5C). In terms of target similarity, we observed that most of the drug combinations worked by targeting different targets (Figure 5A type a). This type of combination generally enhances synergy with complementary mechanisms so often has a more substantial effect. Few cases shared common targets (type b) or targeted the same pathway (type c), resulting in an accumulated effect. Based on drug specificity, most of the drug combinations were combinations of different targeting drugs with high individual specificity (Figure 5B type d). Several cases (type e) used combinations of a highly specific targeting drug and a wide‐spectrum one (e.g., the wide‐spectrum tyrosine kinase inhibitor sunitinib). Combinations of two wide‐spectrum drugs are rare (type f) because of the potential adverse side effects or antagonistic results that might be introduced by broadly targeting diverse proteins. Furthermore, we used metascape^[^ ^45^ ^]^ to enrich the gene function and pathway annotations of the targets in the Kyoto Encyclopedia of Genes and Genomes (KEGG) pathway, hallmark gene sets, biological processes, and molecular functions. Eight primary cancer‐related pathways were enriched (Figure 5C), most of which were related to cell growth and proliferation. As shown by the heatmap, most of the combinations targeted different pathways for synergy, which was consistent with previous analyses. Furthermore, most drugs targeted narrow pathways. In contrast, only wide‐spectrum molecularly targeted drugs, such as sunitinib and dasatinib, exhibited global effects across different pathways by widely inhibiting tyrosine kinases. These analyses provide further insights into selecting drug combinations with specific synergistic mechanisms based on drug targets and pathways.

### 2.5 ScaffComb Enrichment of Synergistic Partner Drugs in Large Databases

Despite the success of the FDA dataset, the number of drugs in this dataset is small. Therefore, to further verify the screening performance on large chemical databases, we applied ScaffComb to the cases discussed in Section 2.1. Therefore, we first used ScaffComb to screen the partner drugs of the FDA‐approved drugs mentioned in Section 2.4 on the ChEMBL database. Similar procedures (Figure 4A) were performed for screening. The major differences were as follows. First, the ChEMBL database was used for screening drugs, which is a far more extensive database (≈870 000) than the FDA dataset. Second, the targets of the screened drugs were identified using the DTI modules shown in Figure 1A. The reference targets for DTI predictions were restricted to the upregulated L1000 landmark genes and their coexpressed L1000 best‐inferred genes in the cell lines.

**Figure 6:**
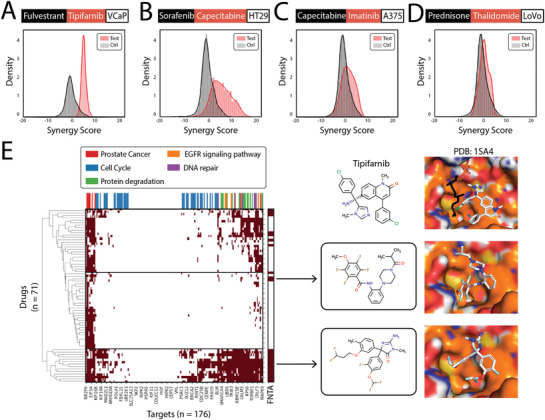
Partner drug screening in the ChEMBL drug database. A–D) Synergy score distributions of screened drugs (red) and randomly selected drugs as a control (gray). Black boxes indicate drug A, red boxes indicate drug B, and white boxes indicate cell lines. A) Fulvestrant‐tipifarnib, a combination of two molecularly targeted drugs. B,C) Capecitabine‐sorafenib and capecitabine‐imatinib, combinations of chemotherapeutic drug and molecularly targeted drugs. D) Prednisone‐thalidomide, a combination of two chemotherapeutic drugs. E) Heatmap of target clustering and pathway annotation in screened drugs from (D) with high synergy scores. Color bar above the heatmap is the pathway annotation. Three compounds are shown on the right, along with binding poses to the FNTA/FNTB complex (PDB ID: 1SA4). Top panels show cocrystal structures of tipifarnib and FNTA/FNTB. Lower two panels show docking poses of two screened compounds predicted to bind to FNTA.

Based on the different mechanisms, we selected four FDA drug combinations in three different situations as examples (Figure S6, Supporting Information). Fulvestrant‐tipifarnib is a combination of two molecularly targeted drugs (**Figure** **6**A and Figure S6A, Supporting Information). The combination of sorafenib‐capecitabine and capecitabine‐imatinib represents the pairing of a chemotherapeutic drug with a molecularly targeted drug (Figure 6B,C and Figure S6B,C, Supporting Information). Prednisone‐thalidomide is a combination of two chemotherapeutic drugs (Figure 6D and Figure S6D, Supporting Information). The basic screening information is summarized in Table S2 in the Supporting Information. For each screening, we compared the synergy score distribution of the screened drugs with randomly chosen drugs (Figure 6A–D). In general, we obtained synergy score enrichment in the screened drugs (Figure 6A–C). This suggests that the use of molecularly targeted drugs in screening could better restrict the space of screened drugs because the phenotypes of molecularly targeted drugs are more specific. In the first screening, we observed that the screened drugs were predicted to target proteins in different mechanism clusters (Figure 6E). In addition to the overall preferences for targeting prostate cancer‐related proteins, the screened drugs also targeted the epidermal growth factor receptor (EGFR) signaling pathway, protein degradation, DNA repair, and cell cycle‐related proteins. In addition, several screened compounds also showed the potential to bind to the protein farnesyltransferase/geranylgeranyltransferase type‐1 subunit alpha (FNTA)/protein farnesyltransferase subunit beta (FNTB) complex (Figure 6E), which was the target of tipifarnib. In contrast, there was no significant synergy score enrichment of the screened drugs during the last screening (Figure 6D). This was mainly due to the fact that the phenotypes of the chemotherapeutic drugs were less specific. The target analyses also indicated that the screened drugs significantly targeted more upregulated proteins than the former (Figure S7A,B, Supporting Information). This suggests that such screening can convey the specificity of the phenotypes; hence, when screening a partner drug, more careful phenotype selection can yield more specific screening results, which reveal the underlying causal links of the biological system.

### 2.6 ScaffComb for Identifying De Novo Drug Combinations and Synergistic Mechanisms in Large Databases

Finally, we used ScaffComb to screen the ZINC database to identify de novo drug combinations in cancer cell lines. The general procedure is shown in Figure S7C in the Supporting Information. Scaffolds were generated using differential gene vectors. We considered two types of phenotypes as the inputs. One was the DEGs between a cancer cell line and the corresponding gene DKO (E3 ubiquitin‐protein ligase (CBL)‐PTPN12 DKO case). The other was the DEGs in a cancer cell line to the corresponding normal tissues (MDA‐MB‐231 case). The downregulated DEGs in DKO and upregulated DEGs in cancer cell lines were considered potential targets for these two cases. Then, we used the scaffolds to screen the ZINC database and obtained drug pairs with the screened compounds. Next, we calculated the synergy scores of the drug pairs and obtained synergistic pairs as candidate drug combinations. Finally, the targets for drugs in the screened drug combinations were analyzed and compared.

**Figure 7:**
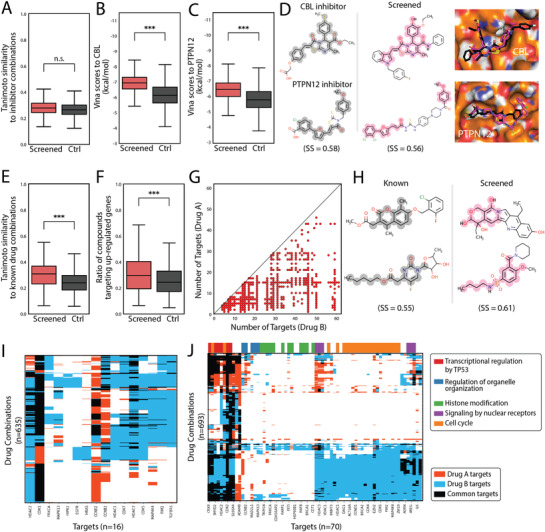
Drug combination screening in ZINC database. A–D) CBL‐PTPN12 double knockout as a phenotype for screening. A) Similarity between inhibitor combinations to screened (red) or randomly chosen (black) drug combinations. n.s.: not significant. B) Binding affinities (smina scores) of screened (red) and control (black) drugs to CBL (PDB ID: 3PLF). C) Binding affinities (smina scores) of screened (red) and control (black) drugs to PTPN12 (PDB ID: 5HDE). *** represents a *p*‐value <0.0001. D) Example of an inhibitor combination that is similar to a screened drug combination. Similar scaffolds are highlighted. Docking poses of compounds are shown on the right. Magenta indicates screened drugs. Black indicates known drugs. E–J) MDA‐MB‐231 cell line and normal breast cell DEGs as the phenotype. E) Similarity between known combinations to screened (red) or randomly chosen (black) drug combinations. F) Ratio of screened (red) and control (black) drugs that target upregulated proteins in MDA‐MB‐231. G) Drug synergistic mechanisms in terms of drug specificity in screened drug combinations with high synergy scores. H) Example of a similar known drug combination and screened drug combination. Similar parts are highlighted. I) Heatmap of screened drug combinations that target known mechanisms. J) Heatmap of screened drug combinations with new mechanisms. Color bar above the heatmap is the pathway annotation.

In the first case, we adopted gene expression of the CBL‐PTPN12 gene double knockout in K562 cell lines from the work of Norman et al.^[^ ^46^ ^]^ The DEGs on L1000 landmark gene sets between DKO and K562 cells were used as the inputs. The screening yielded 20.5 million drug pairs of 6916 compounds in 11 scaffolds, 3062 of which were identified as strong synergistic drug combinations. Twenty‐two inhibitors of CBL and 46 inhibitors of PTPN12 were obtained from ChEMBL. We compared the chemical similarity of the screened drug combinations and the randomly chosen drug combinations to the combinations of the known inhibitors (**Figure** **7**A) but found no significant differences, which might reflect the relatively low chemical diversity of the known inhibitors. However, when we docked the screened drugs to CBL and PTPN12, we observed that the screened drugs had significantly higher binding affinities to the proteins than the random drugs (Figure 7B,C). This suggests that the screened drugs combined with the mechanism of targeting CBL‐PTPN12. An example of a combination of the known inhibitors of the two targets and a screened drug combination exhibited similar scaffolds and similar docking poses (Figure 7D). Furthermore, Figure S7D,E in the Supporting Information shows an example of combinations that were quite different from known inhibitors but with good binding poses and affinities to the two proteins.

In the second case, we used the breast epithelial cell‐derived cancer cell line MDA‐MB‐231 as an example, which was derived from breast epithelial cells. The DEG vector was derived from differences between the cell lines and normal breast cells. The screening yielded 48.8 million drug pairs of 11 253 drugs in 15 scaffolds, 2010 of which were identified as strong synergistic drug combinations. We first compared the chemical similarity of the screened drug combinations and random drug combinations to known effective drug combinations in MDA‐MB‐231 cells. The screened drug combinations were significantly more similar to known drug combinations (Figure 7E). Figure 7H shows an example of screened drug combinations sharing similar scaffolds to known drug combinations. Furthermore, we calculated the ratio of screened drugs that were predicted to target upregulated genes in MDA‐MB‐231, which was significantly higher than that of randomly chosen drugs (Figure 7F). Regarding the drug combination mechanism, the drug combinations in different types of mechanisms were identified (Figure 7G and Figure S7F, Supporting Information), which is consistent with the results shown in Figure 5. This suggested that the phenotype was less specific, because many mechanisms were indicated by the phenotype. However, this type of screening might shed light on the identification of new combination mechanisms. On the one hand, we can identify known combination mechanisms in the screening, such as the combination of CCNE2 and histone deacetylases (HDAC) proteins, mitogen‐activated protein kinase (MAPK), and cyclin‐dependent kinases (CDK) proteins (Figure 7I). On the other hand, novel synergistic combination mechanisms can also be identified (Figure 7J); for example, the combination of SET And MYND Domain Containing 2 (SMYD2) and PRKCA (Protein Kinase C Alpha), in which SMYD2 is related to transcriptional regulation by TP53 and promotes breast cancer,^[^ ^47^ ^]^ whereas PRKCA participates in the regulation of organelle organization and involves several cancers.^[^ ^48^, ^49^ ^]^

These cases suggest that phenotypic specificity can affect the specificity of the screened drugs. However, with fewer specific phenotypes, novel combination mechanisms may be identified. Overall, ScaffComb can identify drug combinations with reasonable mechanisms using different phenotypic constraints.

## 3 Conclusion

In this study, we proposed a phenotype‐based framework for virtual drug combination screening in large chemical databases. First, we described the functional modules of the framework. Specifically, GSG is built to enable the integration of phenotypic information into chemical structures, which is the key aspect restricting screening in large libraries. Additionally, SDSP uses SMILES‐based drug features and a modified network structure to improve drug synergy predictions, enabling ScaffComb to identify potent synergistic drug combinations. We then validated ScaffComb using the FDA‐approved drug dataset and analyzed the preferred synergistic mechanisms in terms of drug targets and pathways. ScaffComb successfully reidentified 65 out of 110 known drug combinations. We also revealed that the most common synergy mechanism in FDA drug combinations is a combination of two molecularly targeted drugs that target different proteins and pathways. Finally, we successfully applied ScaffComb to large database screening. Interestingly, we found that the specificity of phenotypes affects the specificity of the screened drug combinations. High‐specificity phenotypes are suitable for the identification of novel high‐specificity drug combinations. In contrast, general phenotypes may enable the discovery of new synergistic mechanisms.

Thus, ScaffComb is a flexible framework that can incorporate different modules. In this study, we used the generated scaffolds to screen known chemical libraries, i.e., the ZINC and ChEMBL databases. However, we can also generate de novo drug libraries with generated scaffolds using models such as the scaffold decorators described in the work of Arús‐Pous et al.,^[^ ^43^ ^]^ which can be further combined with reinforcement learning^[^ ^36^, ^50^, ^51^ ^]^ for a more property‐focused library design.

A major unsolved problem in our framework is the selection of scaffolds. In this work, we used a modified version of the “rule‐of‐three”^[^ ^52^ ^]^ for filtering scaffolds (see the Experimental Section). However, when a biased generation of molecules is required (e.g., generation of a library where compounds are likely to bind to a specific protein target with high affinity), it was difficult to select scaffolds from simple properties. Therefore, future work should evaluate the criteria for scaffold selection under biased conditions. Alternatively, some end‐to‐end models that can automatically learn scaffold selection rules may also be a viable option. In addition, we used synergy scores to quantify drug effects of drug combinations in this work, which mainly consider the synergistic part of the effects. However, sensitivity is also an important part of drug combinations. Therefore, metrics like the drug combination sensitivity score (CSS),^[^ ^53^ ^]^ which takes both synergy and sensitivity into consideration and can be a better choice for SDSP training in the future.

To the best of our knowledge, ScaffComb is the first framework that performs virtual screening of drug combinations in large chemical libraries and that provides a viable strategy and tool for phenotype‐based drug virtual screening.

## 4 Experimental Section

In this study, data from several public datasets were used. Tokens were restricted to 25 commonly used characters in SMILES, including C, H, O, N, F, S, Cl, Br, c, n, o, s, +, −, 1, 2, 3, 4, 5, 6, = , #, [,], (,). * was used to replace functional groups (R groups) in the scaffolds. < and > were used to mark the initiation and termination tokens in RNN models. In the following preprocessing step, all compounds were filtered using these tokens. The cell lines used in this study included MCF7, MDA‐MB‐231, HS 578T, SK‐MEL‐28, A375, PC‐3, VCaP, A549, SW‐620, HT29, HCT116, LoVo, RKO, and K562 cells.

*Scaffold Generation, Compound Processing, and Docking*: RDKit^[^ ^44^ ^]^ was used to process and visualize compound structures. The scaffold of the compound was extracted using the RECAP algorithm.^[^ ^37^ ^]^ The filtering of scaffolds was set to follow the following rules: 1) it must satisfy the “rule‐of‐three,” 2) rings must be present in scaffolds, 3) fragments with a single ring and a single breakpoint must contain more than ten heavy atoms or two heteroatoms to be considered as scaffolds, and 4) several scaffolds can be generated from a molecule as long as they satisfy (1)–(3). Morgan fingerprints were used to compare the Tanimoto similarity.^[^ ^39^ ^]^ The 3D conformations of a compound were generated using the open babel package.^[^ ^54^ ^]^ Protein 3D structures were downloaded from the research collaboratory for structural bioinformatics (RCSB) PDB database^[^ ^55^ ^]^ (https://www.rcsb.org/). Docking of compounds to proteins was performed with smina^[^ ^41^ ^]^ with an exhaustiveness parameter of 20. All 3D structures were visualized and plotted using the PyMOL 2.4.1.^[^ ^56^ ^]^

*ChEMBL and ZINC Databases*: The ChEMBL 25^[^ ^57^ ^]^ (https://www.ebi.ac.uk/chembl/) dataset was downloaded, and salt and stereochemistry were removed using the RDKit. Duplicate compounds were also removed. Organic compounds shorter than 100 tokens were filtered using the selected tokens and retained. A total of 827 000 unique compounds were retained in the final clean data. Similar processes were also applied to the ZINC database^[^ ^58^ ^]^ (https://zinc.docking.org/), yielding 56 million unique compounds. Target interacting drugs were also extracted from the ChEMBL activity databases with the ChEMBL webresource client^[^ ^59^ ^]^ (https://github.com/chembl/chembl_webresource_client).

*DrugComb Dataset*: The DrugComb^[^ ^15^, ^16^ ^]^ dataset was adopted from https://drugcomb.fimm.fi/. The dataset was filtered using tokens and cell lines. SMILES of Bliss scores were used, and medians were calculated in duplicate samples. Considering the imbalanced distribution of positive and negative samples (Figure 3A), upsampling was applied to augment the positive samples. The original sample distribution was fitted to a normal distribution, *P* *≈* *N*(−0.55, 5.45^2^). A wider distribution *Q* ∼*N* (−0.55, 8.25^2^) was used as the target distribution. 300 000 points were sampled from *Q* and the zone [−50.5, 50.5] was divided into 101 bins. The number of points was then counted in each bin for *P* and *Q* as *n_P_* and *n_Q_*. The |*n_Q_* − *n_P_* | samples were randomly sampled from bins of *P*. As a result, the synergistic and antagonistic samples in *P* were more sampled than samples without obvious effects, which made the three types of samples more balanced. For the up‐sampled samples, the original canonical SMILES strings were transformed to randomized SMILES strings to improve feature extraction and generalization ability when used in RNN;^[^ ^42^, ^43^ ^]^ very minimal normal noise was added to the Bliss scores. As a result, synergistic samples (score ≥5), antagonistic samples (scores ≤ −5), and no‐effect samples (−5 < scores < 5) were relatively balanced (Figure S4B, Supporting Information). Finally, the reversed samples were added to the dataset, meaning that both drugA‐drugB and drugB‐drugA were included in the dataset.

*L1000 Database*: L1000 CMap database level 3 normalized data were downloaded from GSE92742.^[^ ^31^ ^]^ For each sample, 978 landmark genes were identified. Drug‐perturbated and corresponding control samples that were tested with 10 × 10^−6^ m concentration for 6 h in the selected cell lines were retained. Means were calculated for duplicate samples Token filter, salt and stereochemistry removal, length thresholding (≤100), and randomized SMILES strings were applied to the compounds in the samples. Gene expression profiles were min–max normalized to [0,1] as the synergy score prediction inputs. The differentially expressed genes between each drug‐treated sample and the control were set to 1 if the log2 fold change was larger than 1, −1 if the log2 fold change was smaller than −1, or 0 otherwise. After the data were cleaned, 55 000 samples with gene vector‐drug pairs were retained. The gene vector contains 978 L1000 landmark genes in the form of 1, 0, and −1, whereas drugs were represented as SMILES strings. Based on these samples, drugs were further split into scaffolds, yielding 502 000 samples with DEG vector‐scaffold pairs.

For the L1000 gene knockdown data, samples were retained whose corresponding genes had known inhibitors in the ChEMBL databases, yielding 4287 samples. Similar procedures were then performed to process the data.

An extended gene set inferred from the 978 landmark genes was used as a reference target for DTI prediction in each cell line. In the original L1000 work, the authors provided a matrix to infer gene expressions of 21 290 genes from the 978 measured genes, in which 10 874 genes were best inferred. The upregulated DEGs were then chosen in 10 874 genes in cancer cell lines to normal cells as reference targets for DTI predictions.

*BindingDB Database*: The BindingDB^[^ ^60^ ^]^ dataset was downloaded from https://www.bindingdb.org/. According to previous studies,^[^ ^61^ ^]^ samples were first filtered using several criteria. Proteins were restricted to human proteins using UniProt ID. Only drugs with PubChem ID and canonical SMILES lengths shorter than 100 were retained. Experimental values (IC50) smaller than 100 × 10^−9^ m were considered positive samples, whereas values larger than 10 × 10^−6^ m were taken as negative samples. The processing yielded 115 000 positive samples and 133 000 negative samples. In the TransformerCPI dataset construction, the “label reversal experiment” protocol^[^ ^26^ ^]^ was used. Therefore, drugs with at least two interactions were retained, yielding 20 000 training and 5000 testing samples.

*FDA‐Approved Drug Dataset*: The dataset was obtained from the work of Sun et al.^[^ ^62^ ^]^ This dataset includes 61 chemotherapeutic drugs and 89 targeted drugs used in the treatment of 23 cancer types. The SMILES strings of these drugs were obtained from PubChem and cleaned the SMILES strings as previously described. The drug targets were determined from ChEMBL activity data. Synergy scores were obtained using the DrugComb dataset. Finally, 110 FDA‐approved drug combinations were obtained. The details of these drug combinations can be found in the Supporting Information.

For mechanism classification in Figure 5A, if the common target ratio was less than 10% for both drugs, the combination was grouped as type a. If the common target ratio was more than 80% for both drugs, the combination was grouped as type c. Otherwise, the combination was grouped as type b. For mechanism classification in Figure 5B, if a drug had no more than ten targets, it was considered to be a molecularly targeted drug. If a drug had more than 50 targets, it was considered to be a wide‐spectrum drug. The combination of the two molecularly targeted drugs was type d. The combination of the two wide‐spectrum drugs was type e. The combination of a molecularly targeted drug and a wide‐spectrum drug was type f.

*Basal Gene Expression in Normal Tissues and Cancer Cell Lines*: RNA‐seq data for the selected cell lines and corresponding normal tissues (breast, skin, lung, prostate, colon, and bone marrow) were downloaded from the cleaned data in Expression Atlas^[^ ^63^, ^64^ ^]^ (https://www.ebi.ac.uk/gxa/). Specifically, 14 cell line data were from cancer cell line encyclopedia (CCLE);^[^ ^65^ ^]^ six normal tissue and GM12878 data were from encyclopedia of DNA elements (ENCODE).^[^ ^66^, ^67^ ^]^ Bone marrow and GM12878 were used as negative controls for K562 cells. A total of 978 L1000 landmark genes and 9886 best‐inferred genes were retained in the gene expression profiles. MinMax scaling was used to normalize gene expression profiles. Differentially expressed genes were calculated between cell lines and their corresponding normal tissues. The upregulated genes of cell lines in the L1000 best‐inferred gene set were marked as potential targets. Target protein sequences were obtained from the Uniprot database (https://www.uniprot.org/).

*Gene Double Knockout in the K562 Cell Line*: The DKO gene expression data were obtained from the work of Thomas et al.^[^ ^46^ ^]^ in GSE133344, who combined single‐cell RNA‐seq and clustered regularly interspaced short palindromic repeats (CRISPR) screening to study genetic interactions, which provided abundant DKO data in K562 cells. Differential gene vectors were processed in a similar way to that described in the previous section. The downregulated genes of DKO in the L1000 best‐inferred gene set were marked as potential targets. Target protein sequences were obtained from Uniprot (https://www.uniprot.org/).

### GSG

The gene‐scaffold generator followed a seq‐to‐seq with an attention framework, which contained an encoder and an attention decoder (Figure S1A, Supporting Information). The encoder takes the DEG vector ***g*** = (*g*_1_,*g*_2_,*g*_3_,…, *g*_978_) as the input, where *g_i_* ∈ { − 1, 0, 1}. A two‐layer LSTM followed by a fully connected layer to embed *g* was used and it was processed to a context vector ***c*** ∈ $R^{n_{c}\times n_{H}}$, where *n*_C_ and *n*_C_ represent the context dimension and hidden dimension, respectively. In addition to ***c***, during the training time, the inputs of the decoder also contain the drug SMILES ***s =*** (*s*_0_,*s*_2_,…, *s_L_*,*s_T_*), where *s*_0_ = ‘<’ and *s*_T_ = ‘>’ represent initiation and termination tokens. A teacher‐forcing method was used for the training. Therefore, the actual drug input was ***s***_in_ = (*s*_0_,*s*_1_,*s*_2_,..., *s_L_*) and the corresponding label was ***s***_out_ = (*s*_1_,*s*_2_,..., *s_L_*,*s_T_*). ***s***_in_ was first embedded to ***e**_D_*. Then, ***e**_D_* and the previous hidden state ***h*** of the decoder LSTM were used as inputs of a single‐layer neural network Attn( · ) to calculate the importance score ***a*** ∈ $R^{n_{c}}$. The attention weight ***w***_*a*_ ∈ $R^{n_{c}}$ was normalized using ***a*** softmax (Figure S8A, Supporting Information).

(1) $$a=\mathrm{Attn}\left(\left(\mathrm{concat}\right)e_{D},h\right)$$

(2) $$w_{a}^{\left(i\right)}=\frac{\exp\left(a_{i}\right)}{\sum_{j=1}^{n_{c}}\exp\left(a_{j}\right)}$$

Next, ***w***_a_ was used to weight and average across ***c*** and obtain the feature ***F***_*a*_ ∈ $R^{n_{H}}$.

(3) $$F_{a}^{\left(i\right)}=\sum_{j=1}^{n_{C}}w_{a}^{\left(j\right)}c_{i}^{\left(j\right)}$$

Then, the SA‐LSTM is used described in the work of Joulin and Mikolov^[^ ^68^ ^]^ as the scaffold generator in the decoder. ***e***_D_, ***F***_a_, and the previous stack ***s*** ∈ $R^{n_{s}\times n_{H}}$ were used as inputs of the SA‐LSTM (Figure S8B, Supporting Information), where *n*_s_ is the depth of the stack. For the stack part, two single‐layered neural networks, *D*_stack_ and *D*_ctrl_ were used to calculate the stack input vectors ***s***_*stack*_ ∈ $R^{n_{H}}$ and stack control vector ***s***_ctrl_ ∈ {0, 1, −1}^3^ using ***h***.

(4) $$s_{\mathrm{stack}}=\mathrm{Tanh}\left(D_{\mathrm{stack}}\left(h\right)\right)$$

(5) $$s_{\mathrm{ctrl}}=\mathrm{softmax}\left(D_{\mathrm{ctrl}}\left(h\right)\right)$$

Here, ***s***_stack_, ***s***_ctrl_, and ***s*** were used to update the new stack ***s*** ′ using the following equations

(6) $$s^{\prime }\left[0\right]=s_{\mathrm{ctrl}}\left[-1\right]s\left[1\right]+s_{\mathrm{ctrl}}\left[1\right]s_{\mathrm{stack}}+s_{\mathrm{ctrl}}\left[0\right]s\left[0\right]$$

(7) $$s^{\prime }\left[i\right]=s_{\mathrm{ctrl}}\left[-1\right]s\left[i-1\right]+s_{\mathrm{ctrl}}\left[1\right]s\left[i+1\right]+s_{\mathrm{ctrl}}\left[0\right]s\left[i\right]\left(i\in \left[1,n_{s}\right]\right)$$

where *a_t_* [*i*],*i* ∈ {0, 1, −1}the one‐hot vector ***s***_ctrl_ carries the stack operations mentioned in the original paper.^[^ ^68^ ^]^ The RMSprop was used as the optimizer. The learning rate was set to 0.0001 and the batch size was set to 128. The binary crossentropy loss was used during training. To stabilize the training, the SA‐LSTM of the decoder was pretrained first for unconditional scaffold generation on ChEMBL scaffolds (Figure S9A, Supporting Information). Then, the whole model was trained using L1000 gene vector‐scaffold data (Figure S9B, Supporting Information). During training, a decrease in the validity ratio was observed from pretraining; however, the ratio was maintained at a reasonable level. Upon generation time, the gene vector and initiation token were used as inputs. SMILES generation ended when the terminated token was sampled or the length of the generated SMILES was larger than 100.

*SDSP*: The SDSP (Figure S1B, Supporting Information) takes the gene vector *X_G_* = *X_G_* = (*x*_*G*1_,…, *x*_*G*978_) and two drug SMILES*S* = *s*_1_ *s*_2_…*s_L_* (*L* ∈ [10, 100]) as inputs, where *s_i_* ∈ *T* and *T* is a given list of tokens. *S_A_* and *S_B_* were first padded and embedded to process through a two‐layered bidirectional LSTM network followed by one fully connected layer to obtain drug embeddings ***z***_*A*_ ∈ $R^{n_{H}}$ and ***z***_*B*_ ∈ $R^{n_{H}}$, where *n*_H_ represents the hidden dimension. *X*_G_ was embedded in a two‐layered fully connected network to obtain gene embedding ***z***_*G*_ ∈ $R^{n_{H}}$. Then, the embeddings were concatenated to obtain ***z**_AB_* and ***z**_BA_*.

(8) $$z_{AB}=\mathrm{concat}\left(z_{G},z_{A},z_{B}\right)$$

(9) $$z_{BA}=\mathrm{concat}\left(z_{G},z_{B},z_{A}\right)$$

Two three‐layered fully connected networks were used to predict the synergy scores *ss_AB_* and,*ss_BA_* respectively, using concatenated embeddings. The final prediction $\hat{ss}$ was the mean of the two scores.

(10) $$\hat{ss}=\frac{ss_{AB}+ss_{BA}}{2}$$

The total loss contains the mean squared error loss between the predicted and true values and the mean absolute error (MAE) loss that penetrates the differences between the AB and BA predictions. *α* was set to 1.

(11) $$L=\mathrm{MSE}\left(ss,\hat{ss}\right)+\alpha \mathrm{MAE}\left(ss_{AB},ss_{BA}\right)$$

Adam was used as an optimizer. The learning rate was set to 0.0001, the batch size was set to 256, and the gradient was updated every ten batches. Upsampling of positive samples was applied to the training set. Randomized SMILES was used for both the training and test sets. The DSP is similar to the SDSP in structure, but uses chemical fingerprints as drug features instead of SMILES strings. Therefore, the LSTMs were replaced with two‐layered fully connected networks in the SDSP. The changes in the losses of the training and test sets are shown in Figure S9C in the Supporting Information.

*TransformerCPI*: Drug‐protein interactions were predicted using the reported TransformerCPI^[^ ^26^ ^]^ (Figure S1C, Supporting Information). TransformerCPI is a classifier that takes the drug SMILES and target protein sequences as inputs. SMILES are processed with a graph convolution neural network^[^ ^69^ ^]^ to obtain drug embeddings ***z***_*D*_ ∈ $R^{n_{H}}$. Protein sequences were first condensed with a pretrained word2vec model to a fixed‐length vector, which was then processed with a gated convolutional network to obtain protein embedding ***z***_*P*_ ∈ $R^{n_{H}}$. Then, taking the embeddings, the transformer decoder uses a multiheaded self‐attention layer to extract interaction information and obtain an interaction feature vector ***z*** ∈ $R^{n_{H}}$, which is fed to a fully connected network for the output *y_i_* ∈ {0, 1}. Binary crossentropy loss was used for the classification task.

(12) $$L=-\sum_{i=1}^{N}y_{i}\log{\hat{y}}_{i}+\left(1-y_{i}\right)\log\left(1-{\hat{y}}_{i}\right)$$

Hyperparameters were set as recommended in the original study. The model was trained on the BindingDB dataset using a “label reversal experiment” protocol for training test set preparation.

The augmented DSP was compared with several baseline and state‐of‐the‐art methods. The DrugComb dataset was used to train and evaluate the methods. Fivefold crossvalidation was used. The training and testing sets were split into unique drug pairs. ECFP6 fingerprints or SMILES were used as the chemical descriptors for drugs, whereas basal gene expression of L1000 landmark genes was used as the cell line feature. Elastic net,^[^ ^70^ ^]^ random forest,^[^ ^71^ ^]^ and XGBoost^[^ ^72^ ^]^ models were built and trained using the scikit‐learn package.^[^ ^73^ ^]^ DeepSynergy,^[^ ^17^ ^]^ MatchMaker,^[^ ^19^ ^]^ and DSP and SDSP models were built and trained using PyTorch.^[^ ^74^ ^]^ All models except SDSP used two drug ECFP6 fingerprints and L1000 cell line basal gene expression as inputs. SDSP used two drugs, SMILES, and L1000 cell line basal gene expression as inputs. Elastic nets are linear models with a feature selection. Random forest and XGBoost were tree‐based nonlinear models. DeepSynergy used a fully connected deep neural network structure. MatchMaker combined individual drug features with cell line features and concatenated the two processed features to predict drug synergy in an end‐to‐end neural network structure. Details of DSP and SDSP are described in SDSP section. Models were tested on regression, classification, and AB‐BA correlation tasks. In the classification task, samples with Bliss scores >5 (synergistic) or < −5 (antagonistic) were considered positive; all others were considered negative samples.

## Conflict of Interest

The authors declare no conflict of interest.

## Author Contributions

The author contribution is as follows: Conceptualization (Z.Y., J.G., and M.Q.Z.); Methodology (Z.Y.); Investigation (Z.Y. and F.C.); Visualization (J.G. and Z.Y.); Supervision (J.G., J.Z., and M.Q.Z.); Writing‐original draft (Z.Y.); Writing‐review and editing (all).

## Supporting information

Supporting Information

## Data Availability

The data that support the findings of this study are available from the corresponding author upon reasonable request.
