# Supplementary material for: ScaffComb: A Phenotype‐Based Framework for Drug Combination Virtual Screening in Large‐Scale Chemical Datasets
Source: Adv Sci (Weinh). 2021 Nov 1;8(24):2102092. doi: 10.1002/advs.202102092 (PMC8693048; doi:10.1002/advs.202102092)
Supplement: Supplementary file 1 — Supporting Information [file ADVS-8-2102092-s001.pdf]

© 2021 Wiley-VCH GmbH

ADVANCED SCIENCE
Open Access

Supporting Information

for *Adv. Sci.*, DOI: 10.1002/advs.202102092

ScaffComb: A Phenotype-Based Framework for Drug Combination Virtual Screening in Large-scale Chemical Datasets

Zhaofeng Ye[1, 2], Fengling Chen[3, 4], Minglei Shi[1, 2], Jiangyang Zeng[2, 5], Juntao Gao[2] and Michael Q. Zhang[1, 2, 6]**

Supplementary Materials

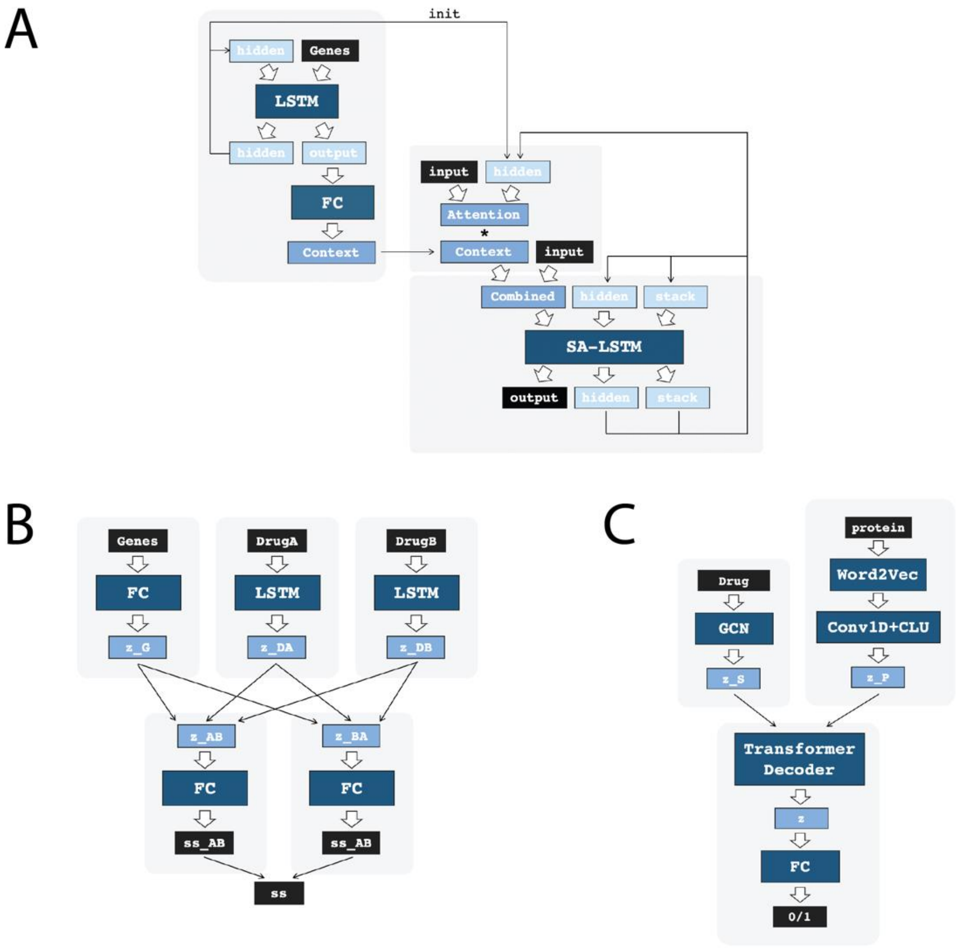

Figure S1. Structure of modules in ScaffComb. A. Gene-scaffold generator, which takes differential gene expression vectors as the input to generate scaffolds. **B.** Drug synergy predictor, which takes the basal expression of cell lines and two drug SMILES as inputs to calculate synergy scores. **C.** Drug-target interaction classifier; TransformerCPI was used in this work. The classifier was trained to predict drug-target interactions. FC: fully connected layers. LSTM: long short-term memory. SA-LSTM: stack-augmented LSTM. GCN: graph convolutional network. GLU: gated linear unit.

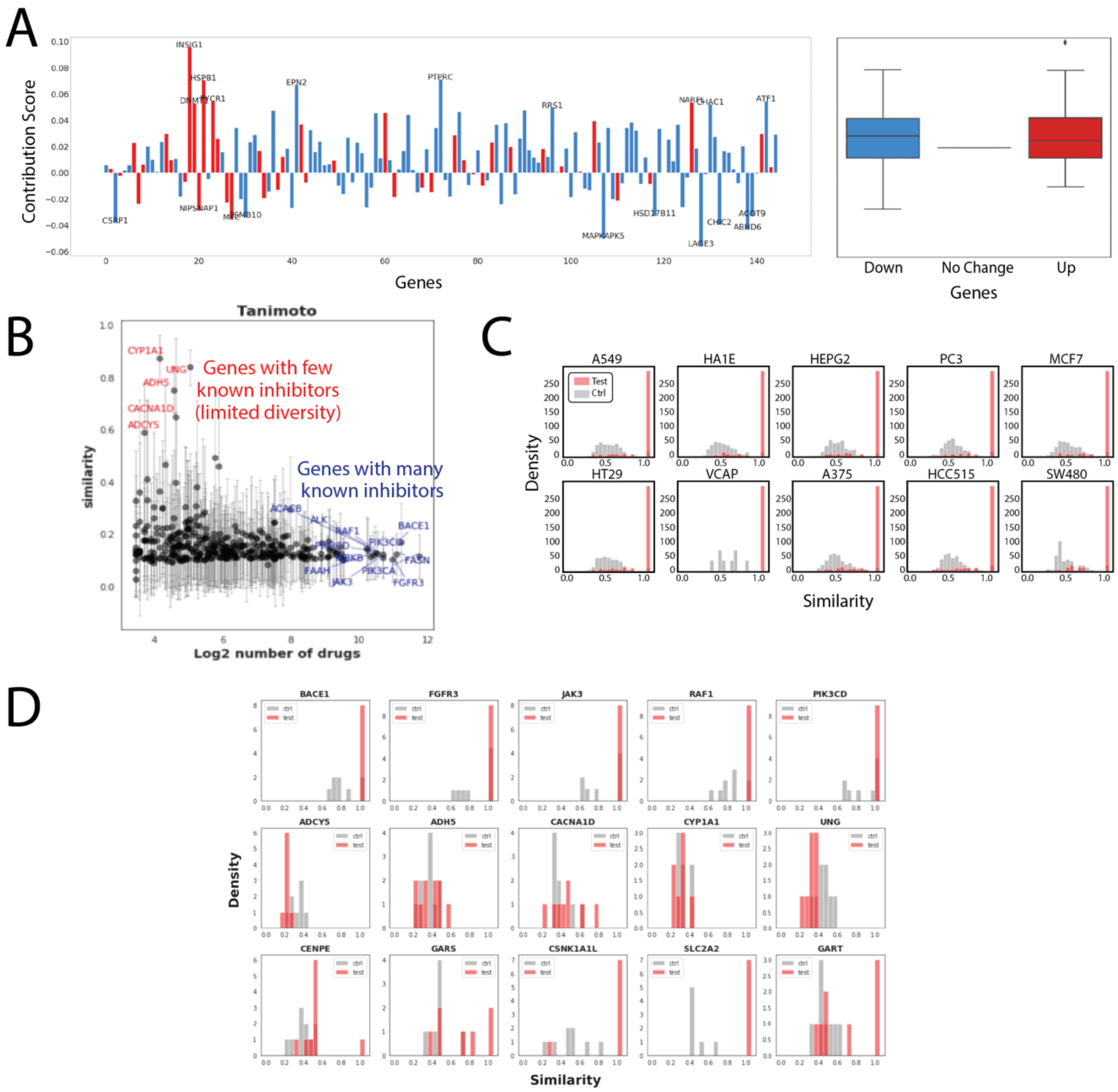

Figure S2. Performance evaluations for the gene-scaffold generator. A. An example of the contribution of upregulated and downregulated genes in the input gene signatures to the scaffold generation. **B.** Number of inhibitors and similarity of intra-target inhibitor scaffolds. Red represents genes with few inhibitors but high similarity. Blue represents genes with many inhibitors. **C.** Similarity distribution comparison of inhibitor scaffolds to generated scaffolds (red) and random scaffolds (gray) in the different cell lines. **D.** Sampled targets for scaffold generation comparison. Upper panels show blue targets; middle panels show red targets; lower panels show randomly selected black targets

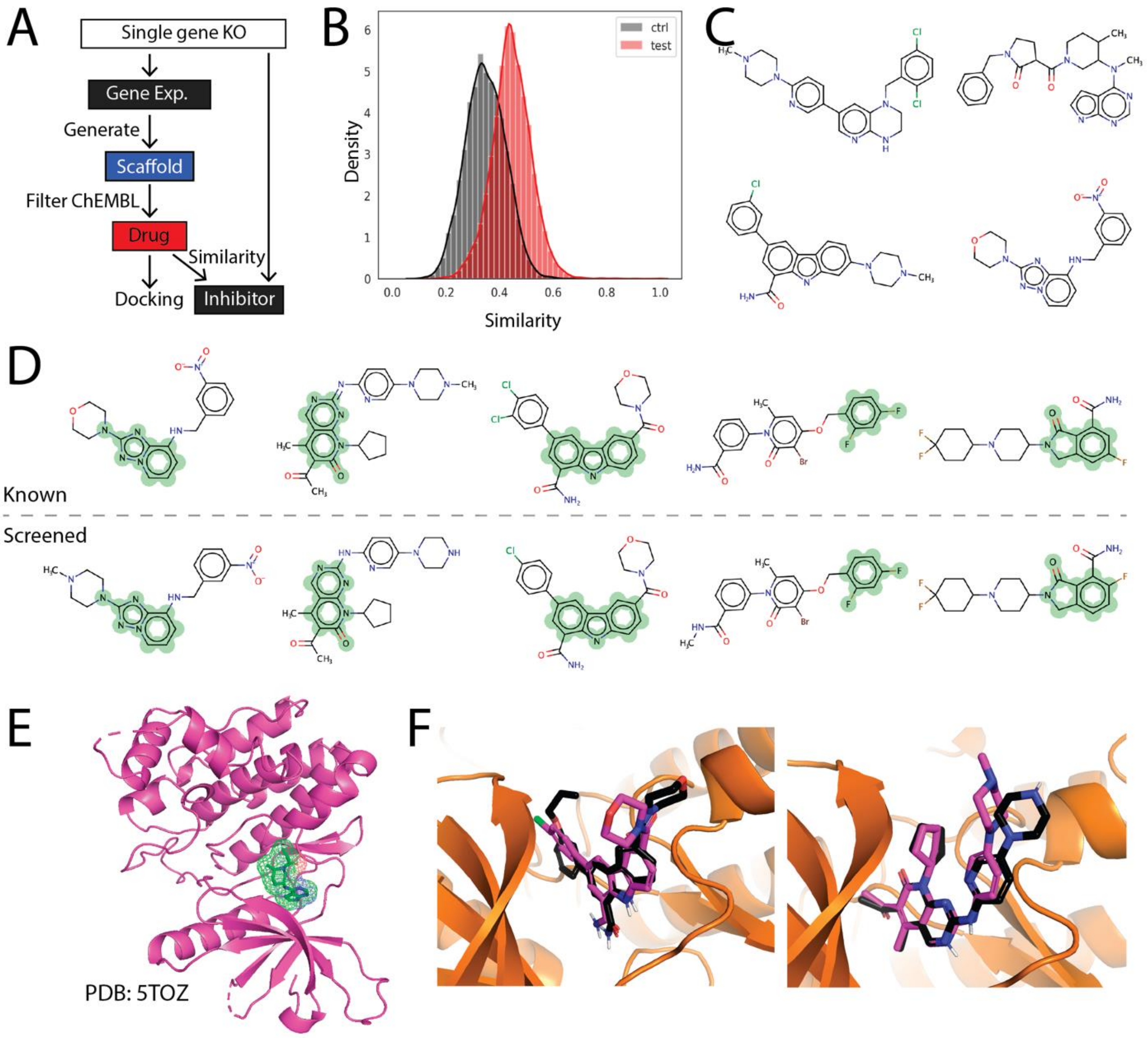

Figure S3. Screening results based on the JAK3 knockout phenotype in ChEMBL. A. Pipeline for drug screening. **B.** Similarity of known inhibitors to screened drugs (red) and random drugs (black). **C.** Some known inhibitors were screened. **D.** Some compounds with high similarity to known inhibitors were screened. **E.** Crystal structure of the protein kinase domain of JAK3 (PDB ID: 5TOZ). Green meshes show a binding compound. **F.** Some binding poses of screened compounds (red) and known inhibitors (black) to JAK3. Orange represents protein structures.

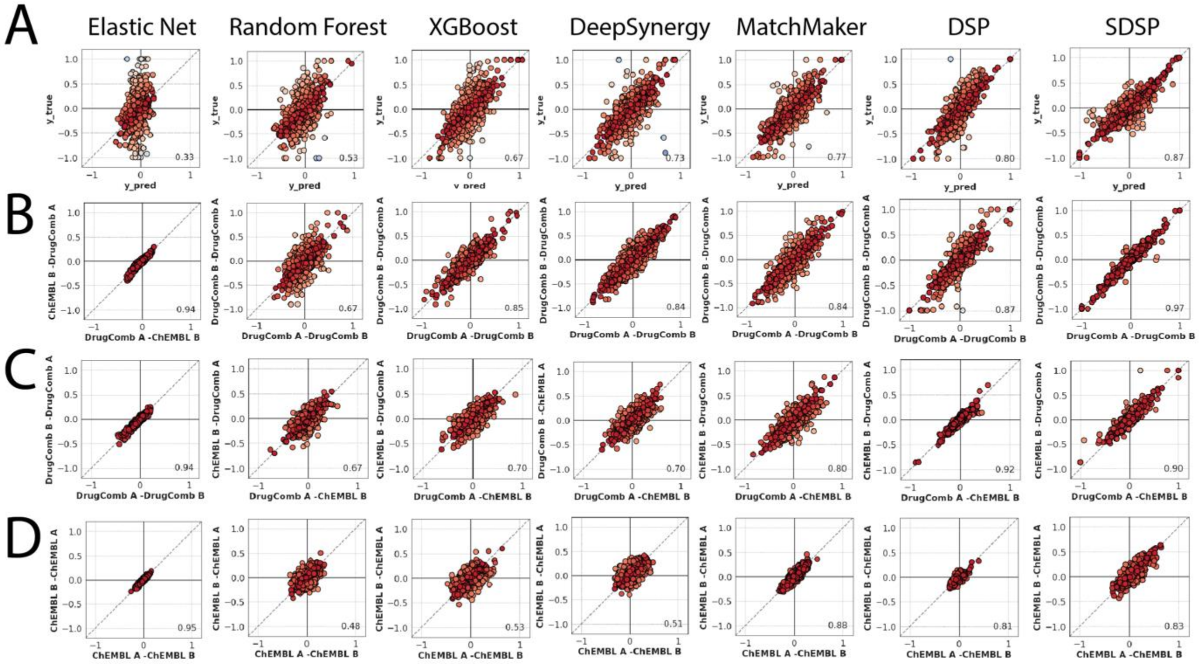

Figure S4. Performances for different models in regression tasks. A. Performance on test set. **B–D.** AB-BA correlation of different methods using different sources of compounds. **B.** Two drugs from DrugComb. **C.** One from DrugComb and one drug from ChEMBL. **D.** Two drugs from ChEMBL.

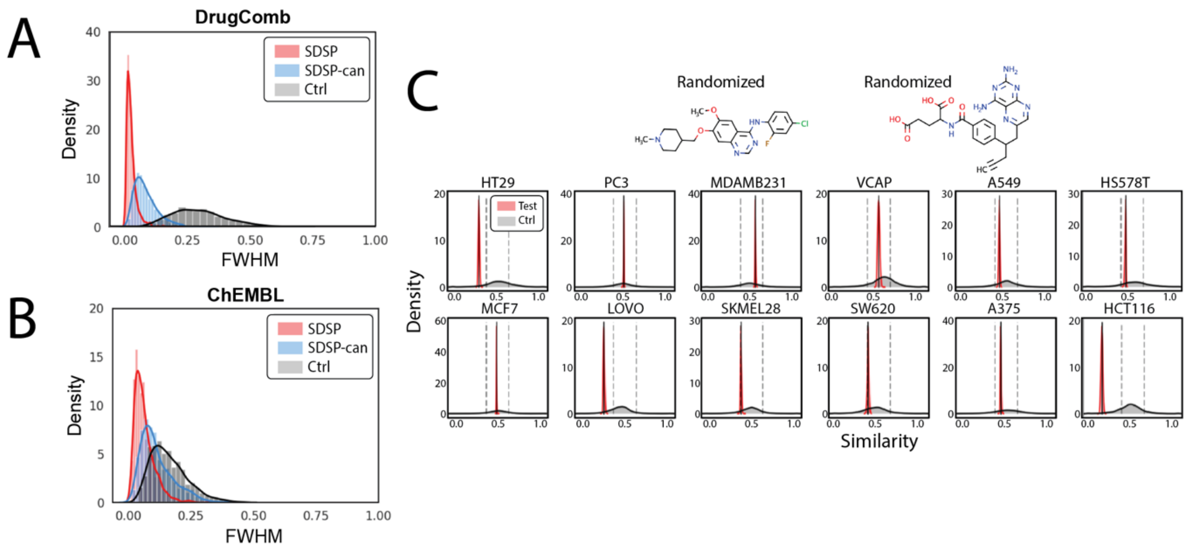

Figure S5. SMILES-based drug synergy predictor predicts consistent results for different forms of SMILES. A, B. FWHM distributions of synergy scores using randomized SMILES strings from DrugComb (**A**) or ChEMBL (**B**) as inputs. Red represents predictions with SDSP. Blue represents predictions with SDSP-can. Gray represents random control. **C.** An example of the distribution of synergy scores with randomized SMILES strings using SDSP (red) and synergy scores between random controls (gray).

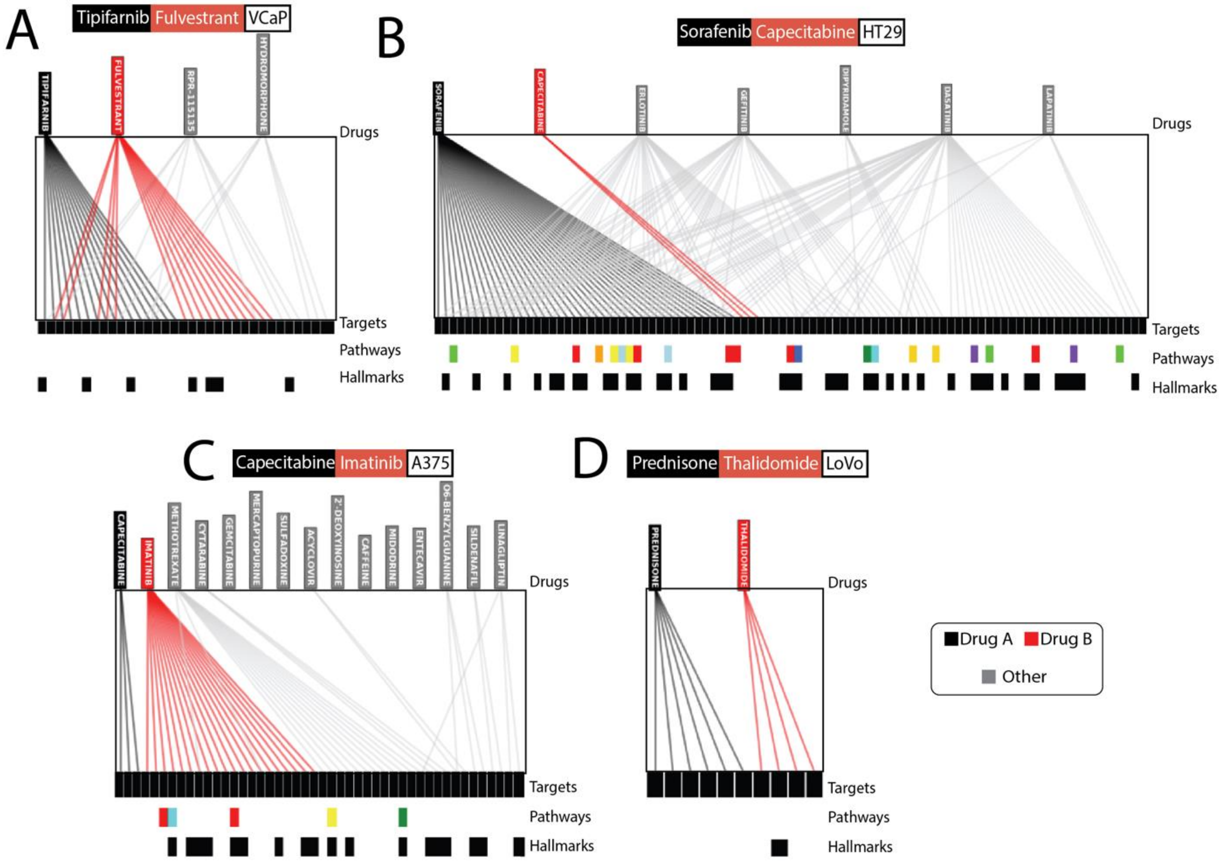

Figure S6. Diverse combination mechanism in FDA-approved drug combinations. A. Combination of two chemotherapeutic drugs. **B, C.** Combinations of a chemotherapeutic drug and a targeted drug. **D.** Combination of two targeted drugs. Upper boxes show drug combination components. Lower color bars show enriched pathways. Lower black bars shown hallmark genes in cancer.

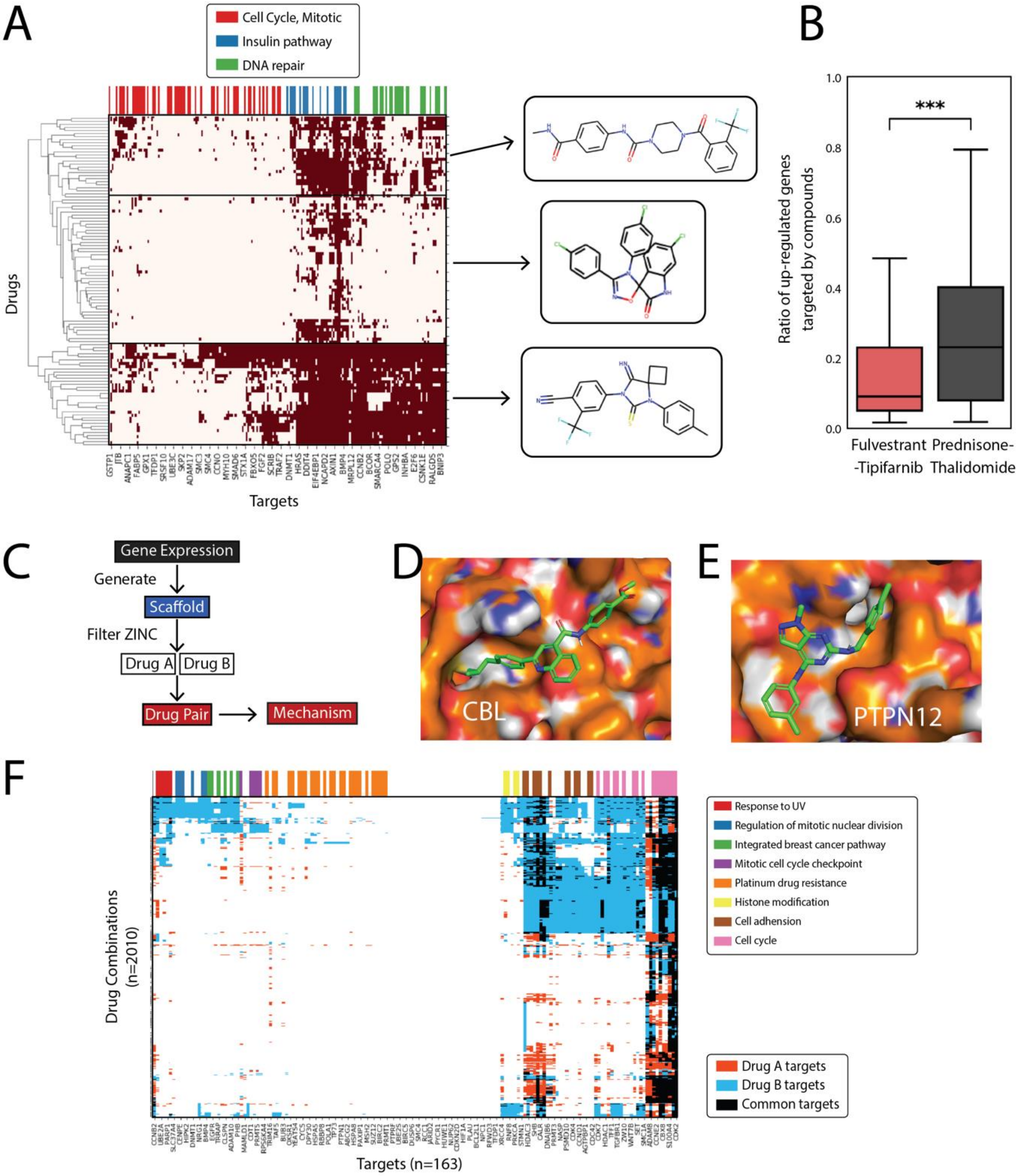

Figure S7. Drug combination screening of large chemical libraries using ScaffComb. A. Heatmap of screened combined drugs in the prednisone-thalidomide case. Top bars show enriched pathways. Boxes show chemical structures of middle points of the clusters. **B.** Ratio of upregulated genes targeted by screened drugs in the fulvestrant-tipifarnib case (red) and prednisone-thalidomide case (black). ***

indicates a p-value < 0.0001 **C.** Pipeline for screening drug combinations from some phenotypes. **D, E.** Binding poses of some screened compounds to CBL and PTPN12 that are dissimilar to known inhibitors. Green represents chemical structures. Orange represents protein structures. **F.** Heatmap of combination mechanisms of screened drug combinations with high synergy scores in the MDA-MB-231 cell line. Red represents targets for drug A; blue represents targets of drug B; black represents common targets. Top color bar shows the enriched pathways.

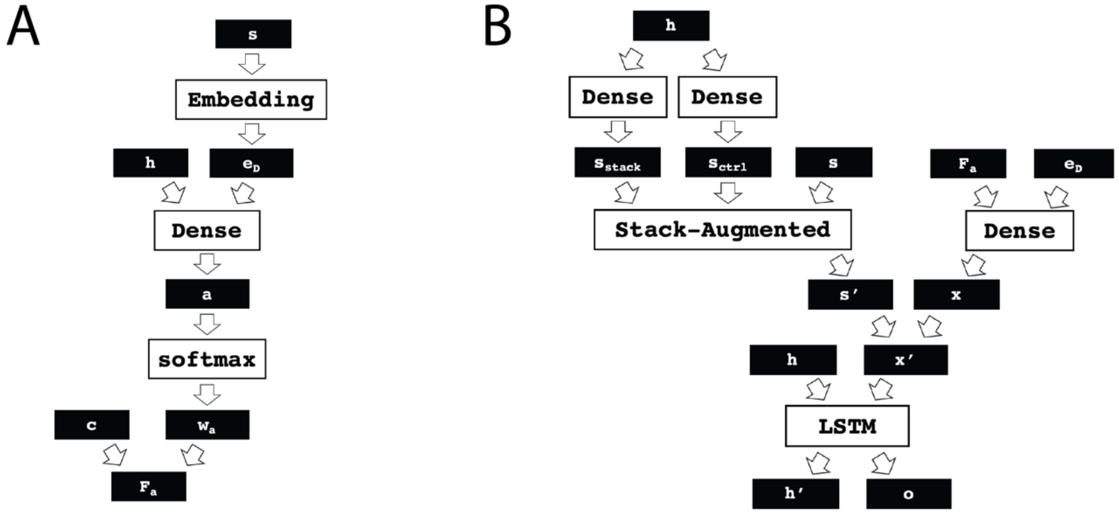

Figure S8. Network details of GSG attention module and SA-LSTM module. A. Attention module. **B.** SA-LSTM module. Black boxes indicate input, intermediate, or output vectors. White boxes indicate neural networks.

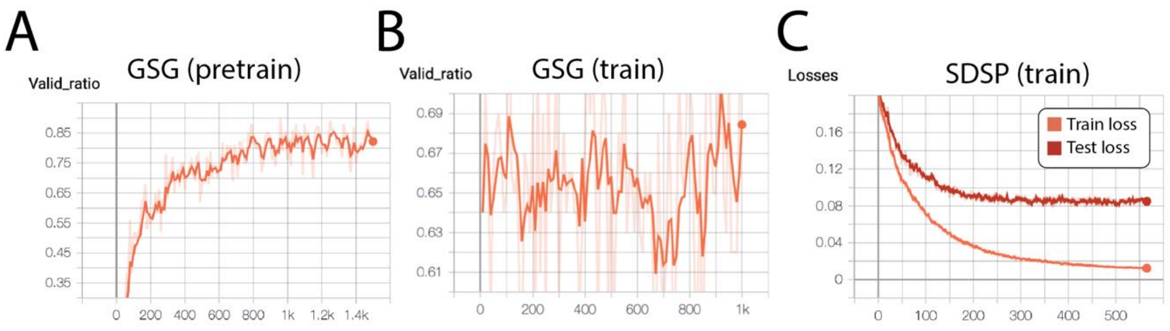

Figure S9. Training processes of GSG and SDSP. A, B. Validity ratio changes during pre-training and training phases for GSG. **A.** GSG was pre-trained with ChEMBL scaffolds. **B.** GSG was then trained with L1000 scaffolds along with DEG vectors. **C.** Train and test loss changes during SDSP training.

Table S1 Synergy score prediction performance across different methods

**Method** [a]	**Regression**		**Classification** [b]		**AB-BA correlation** [c]		
	Spearman ρ	Pearson r	AUC	AUPRC	DrugComb-DrugComb	DrugComb-ChEMBL	ChEMBL-ChEMBL
**Elastic Net**	0.33 ± 0.01	0.34 ± 0.01	0.66 ± 0.02	0.28 ± 0.04	0.94 ± 0.01	**0.94 ± 0.01**	**0.96 ± 0.02**
**Random Forest**	0.52 ± 0.02	0.58 ± 0.02	0.75 ± 0.01	0.47 ± 0.03	0.67 ± 0.01	0.67 ± 0.02	0.47 ± 0.03
**XGBoost**	0.61 ± 0.02	0.67 ± 0.01	0.80 ± 0.02	0.51 ± 0.04	0.85 ± 0.02	0.69 ± 0.02	0.50 ± 0.06
**DeepSynergy**	0.68 ± 0.05	0.74 ± 0.06	0.85 ± 0.01	0.64 ± 0.03	0.85 ± 0.01	0.70 ± 0.01	0.50 ± 0.02
**MatchMaker**	0.72 ± 0.04	0.77 ± 0.02	0.86 ± 0.04	0.68 ± 0.04	0.84 ± 0.01	0.80 ± 0.02	0.88 ± 0.01
**DSP**	0.76 ± 0.04	0.80 ± 0.02	0.88 ± 0.01	0.71 ± 0.03	0.87 ± 0.01	0.92 ± 0.01	0.81 ± 0.01
**Augmented DSP**	**0.80 ± 0.04**	**0.85 ± 0.02**	**0.90 ± 0.01**	**0.80 ± 0.04**	**0.97 ± 0.01**	0.90 ± 0.01	0.82 ± 0.01

[a] Input features were gene expression signatures as cell line features and ECFP4 fingerprints as drug features for all methods except SDSP, which used randomized SMILES strings as drug features.
[b] Samples with Bliss scores > 5 are considered positive, whereas values < -5 are considered negative. AUC: area under the ROC curve; AUPRC: area under the precision-recall curve.
[c] Mean and standard deviation of 1,000 combinations in different cell lines.

Table S2 Combined drug screening in the ChEMBL database

Combination [a]	Phenotype	No. scaffolds for screening	No. screened drugs [b]	No. positive drugs [c]
Prednisone-Thalidomide-LoVo	Thalidomide	8	90,870	602
Sunitinib-Capecitabine-HT29	Capecitabine	3	3,366	1,393
Tipifarnib-Fulvestrant-VCaP	Fulvestrant	2	4,060	480
Capecitabine-Imatinib-A375	Imatinib	7	16,493	1,015

[a] Drug A - Drug B - Cell line

[b] Filtered ChEMBL database contains more than 870,000 drugs.

[c] Drug combinations with Bliss synergy scores > 5 or < -5 were considered positive.
